# Trends in thyroid function testing, neck ultrasound, thyroid fine needle aspiration, and thyroidectomies in North-eastern Italy

**DOI:** 10.1007/s40618-020-01475-3

**Published:** 2021-01-18

**Authors:** L. Dal Maso, C. Panato, A. De Paoli, V. Mattioli, D. Serraino, R. Elisei, G. Zoppini, C. Gobitti, E. Borsatti, E. Di Felice, F. Falcini, S. Ferretti, S. Francisci, P. Giorgi Rossi, S. Guzzinati, G. Mazzoleni, D. Pierannunzio, S. Piffer, S. Vaccarella, M. Vicentini, M. Zorzi, S. Franceschi, U. Fedeli, for the DEPTH Working Group, F. Avossa, R. De Palma, R. Vattiato, A. Polverino, F. Vittadello, F. Toffolutti, G. Fanetti

**Affiliations:** 1 Cancer Epidemiology Unit, Centro di Riferimento Oncologico di Aviano (CRO) IRCCS, Via Franco Gallini 2, 33081 Aviano, PN Italy; 2 Epidemiological Department, Azienda Zero, Via J. Avanzo, 35, 35132 Padua, Italy; 3 Unit of Endocrinology, Department of Clinical and Experimental Medicine, University Hospital of Pisa, Pisa, Italy; 4 Endocrinology, Diabetes and Metabolism, Department of Medicine, University and Hospital Trust of Verona, Verona, Italy; 5 Radiation Oncology, Centro di Riferimento Oncologico di Aviano (CRO), IRCCS, 33081 Aviano, Italy; 6 Nuclear Medicine Unit, CRO Aviano National Cancer Institute, Via Franco Gallini, 2, 33081 Aviano, Italy; 7 Authority for Healthcare and Welfare, Emilia Romagna Regional Health Service, Bologna, Italy; 8 Romagna Cancer Registry, Istituto Scientifico Romagnolo per lo Studio e la Cura dei Tumori (IRST), IRCCS, Meldola, Italy; 9 Azienda Usl della Romagna, Forlì, Italy; 10 Ferrara Cancer Registry, University of Ferrara, Azienda USL Ferrara, Ferrara, Italy; 11 National Centre for Disease Prevention and Health Promotion, National Institute of Health, Rome, Italy; 12 Reggio Emilia Cancer Registry, Epidemiology Unit, AUSL ASMN-IRCCS, Azienda USL di Reggio Emilia, Reggio Emilia, Italy; 13 South Tyrol Cancer Registry, Bolzano, Italy; 14 Trento Province Cancer Registry, Unit of Clinical Epidemiology, Trento, Italy; 15 Section of Cancer Surveillance, International Agency for Research on Cancer, Lyon, France

**Keywords:** Thyroid stimulation hormones (TSH), Neck ultrasound, Fine needle aspiration, Thyroidectomies, Italy

## Abstract

**Purpose:**

Evidence of an increased diagnostic pressure on thyroid has emerged over the past decades. This study aimed to provide estimates of a wide spectrum of surveillance indicators for thyroid dysfunctions and diseases in Italy.

**Methods:**

A population-based study was conducted in North-eastern Italy, including 11.7 million residents (20% of the total Italian population). Prescriptions for TSH testing, neck ultrasound or thyroid fine needle aspiration (FNA), surgical procedures, and drugs for hypo- or hyperthyroidism were extracted from regional health databases. Proportions and rates of selected examinations were calculated from 2010 to 2017, overall and by sex, calendar years, age, and region*.*

**Results:**

Between 2010 and 2017 in North-eastern Italy, 24.5% of women and 9.8% of men received at least one TSH test yearly. In 2017, 7.1% of women and 1.5% of men were prescribed drugs for thyroid dysfunction, 94.6% of whom for hypothyroidism. Neck ultrasound examinations were performed yearly in 6.9% of women and 4.6% of men, with a nearly two-fold variation between areas. Thyroid FNA and thyroidectomies were three-fold more frequent in women (394 and 85 per 100,000) than in men (128 and 29 per 100,000) with a marked variation between areas. Both procedures decreased consistently after 2013.

**Conclusions:**

The results of this population-based study describe recent variations over time and between surrounding areas of indicators of ‘diagnostic pressure’ on thyroid in North-eastern Italy. These results emphasize the need to harmonize practices and to reduce some procedures (e.g., neck ultrasound and total thyroidectomies) in certain areas.

**Supplementary Information:**

The online version contains supplementary material available at 10.1007/s40618-020-01475-3.

## Introduction

Thyroid diseases can be broadly summarized into thyroid dysfunctions (hypothyroidism, hyperthyroidism) and structural diseases (goiter, benign nodules, and cancer). An increased frequency of thyroid diseases has been observed over the past three decades [1–4].

National and international guidelines and recommendations about the management of thyroid diseases are available [5–7], but an abuse of testing and diagnostic procedures may lead to wasting of resources, overdiagnosis, and inappropriate treatment [8]. In addition, still open issues concern whether healthy adults can benefit from screening for thyroid diseases, including targeted screening for thyroid dysfunction in any circumstance, including pregnancy [4] and low-radiation exposures [9]. Indeed, the assessment of the thyroid function has become common practice, in particular thyroid function testing through TSH (i.e., the most sensitive marker of thyroid status) that is increasingly included among routine tests for metabolic status or in the presence of a broad range of medical conditions [10, 11]. Concurrently, a massive increase in the neck ultrasound to explore the thyroid gland or other abnormalities of the neck has been reported in many regions of the world [12, 13], and intensity of medical surveillance has been shown to be a major determinant of the large, although heterogeneous, rise in thyroid cancer incidence over the past three decades [8, 13–22]. In particular, a very frequent detection of asymptomatic tumors, which may have never caused symptoms or harm, emerged. In addition, an increased search for cancers (incidental microcarcinomas) in patients undergoing surgery for benign thyroid diseases has been observed [12, 23–25].

Given the high and increasing thyroid cancer incidence reported in Italy [26], with progressively increasing geographic variation [27], and paucity of population-based studies describing the changes over time of ‘diagnostic pressure’ on thyroid [12, 22, 24, 28–31], this study aimed to provide population-based estimates of the frequency and trends of a wide spectrum of medical surveillance indicators for thyroid dysfunction and disease in North-eastern Italy. We explored the variations of thyroid function testing, chronic treatments, neck ultrasound, thyroid fine needle aspiration (FNA), frequency and type of thyroid surgery by sex, over time, and between areas.

## Materials and methods

This population-based study was conducted to identify the frequency of thyroid examinations in North-eastern Italy, three administrative regions—Veneto, Emilia-Romagna, and Friuli Venezia Giulia—and two autonomous provinces—Trentino and Alto Adige (Table 1), for a total of 11.7 million residents in 2017 (20% of the total Italian population, 5.7 million men and 6.0 million women). Well comparable and comprehensive regional health system databases are active in this part of Italy, including exhaustive information on medical prescriptions and procedures since the 1990s, and regional population-based cancer registries [26]. The following databases collected by regional health authorities were examined: outpatient services databases; hospital discharge databases; and drug prescriptions databases (Online Appendix 1). Prescriptions for TSH testing (ICD9-CM code 90.42.1) [32], neck ultrasound examinations (ICD9-CM codes 88.71.4, 88.71.5, 88.73.5); and thyroid FNA (i.e., FNA biopsy or FNA cytology, ICD9-CM codes 06.01, 06.01.1, 06.11.1, 06.11.2) were retrieved from outpatient services databases in each year from 2010 to 2017 by area, sex, and age.

**Table 1:** Regions included in the study, population, and proportion of people who underwent selected thyroid examinations at least once in any year. North-eastern Italy, 2010–2017

	Population		Drugs for hypo-hyperthyroidism^a^		TSH		Neck ultrasound		Thyroid fine needle aspiration		Thyroidectomies		Thyroid cancer^b^	
	Million	Mean	Per 100						Per 100,000					
		Age^c^												
		2017	2017		2010–2017^d^		2010–2017^d^		2010–2017^d^		2010–2017^d^		2010–2012^d^	
			Men	Women	Men	Women	Men	Women	Men	Women	Men	Women	Men	Women
Region														
Total	11.65	45.5	1.5	7.1	9.8	24.5	4.6	6.9	128	394	29	85	10	21
Veneto	4.91	45.1	1.1	5.5	8.7	22.6	4.3	6.1	106	327	28	86	8	21
Emilia-Romagna (ER)	4.46	45.9	1.8	8.5	11.2	27.0	5.2	8.2	172	536	35	95	15	24
Friuli Venezia Giulia (FVG)	1.22	47.1	1.5	7.9	8.1	21.5	3.7	5.4	107	354	21	65	7	18
Trentino	0.54	44.2	1.6	8.2	10.8	31.0	5.6	8.2	71	205	16	49	6	18
Alto Adige/South Tyrol (AA)	0.53	42.5	1.5	6.7	10.6	22.3	2.9	4.3	70	78	27	77	5	11

^a^At least two drug prescriptions in 2017

^b^Adapted from Dal Maso et al. [26], age 0–84 years

^c^http://dati.istat.it/Index.aspx?DataSetCode=DCIS_INDDEMOG1#

^d^Average number of people undergoing each test or procedure at least once per year

Surgical procedures, including partial (ICD9-CM codes 06.2, 06.3x, 06.51) [33] and total (ICD9-CM codes 06.4, 06.50, 06.52) thyroidectomies for any indication were extracted from hospital discharge databases for each year between 2010 and 2017 by area, sex, and age. The number of FNA was also extracted from Hospital Discharge databases. For each individual only one procedure of TSH testing, neck ultrasound, thyroid FNA, and thyroidectomy per year was counted.

In order to characterize the prevalence of people living with “chronic” thyroid dysfunction, including hyperthyroidism or hypothyroidism (for all reasons, including thyroidectomies), individuals with at least two prescriptions for thyroid (ATC code H03AA) [34] and anti-thyroid (code H03BB) preparations were identified in 2017 from the drug prescriptions databases.

On account of the descriptive purpose of the present research and the demographic similarities of the examined regions, we computed only crude proportions and rates of selected examinations from 2010 to 2017, overall and by sex, calendar years, age, and region*.*

## Results

**Fig. 1:**
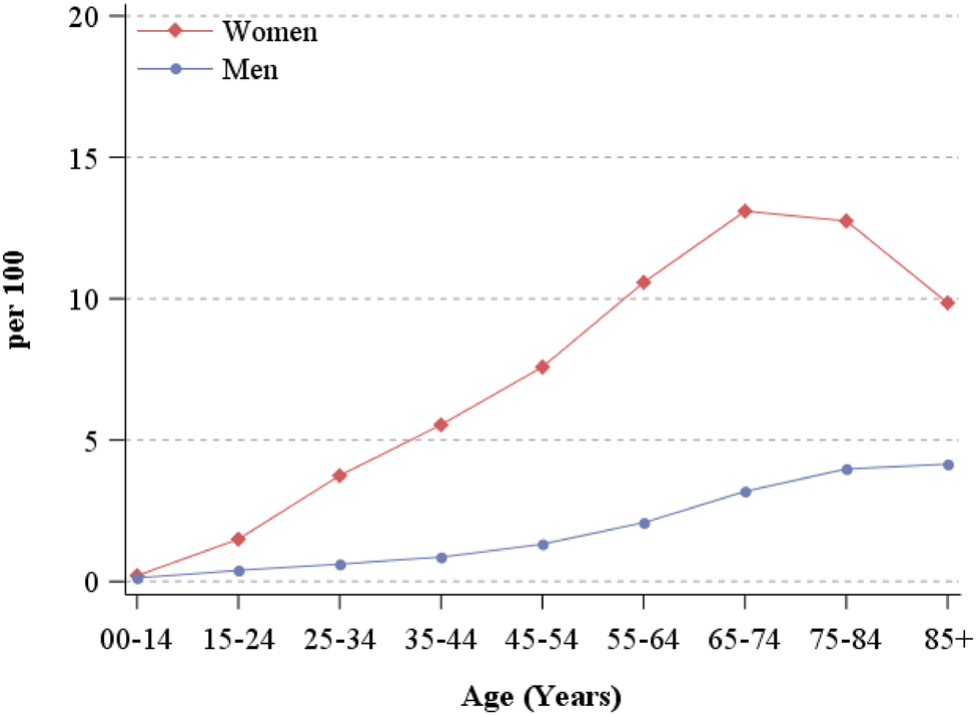
People to whom drugs for hypothyroidism or hyperthyroidism were prescribed at least twice in 2017, by sex and age group. North-eastern Italy

In 2017, 7.1% of women and 1.5% of men living in North-eastern Italy were prescribed, at least twice, drugs for hypothyroidism or hyperthyroidism (Table 1). Drugs use increased linearly with age until 65–74 years in women (13.1%) and 75 years or older in men (4.0%) (Fig. 1). The vast majority (94.6%) of these prescriptions were thyroid hormones (mainly levotiroxine) for hypothyroidism, the remaining 5.4% were thyroid inhibitors (mainly thiamazole, also known as methimazole) used to treat hyperthyroidism. The highest proportions of drugs for hypo/hyperthyroidism emerged in Emilia Romagna (8.5% in women and 1.8% in men) (Table 1).

**Fig. 2:**
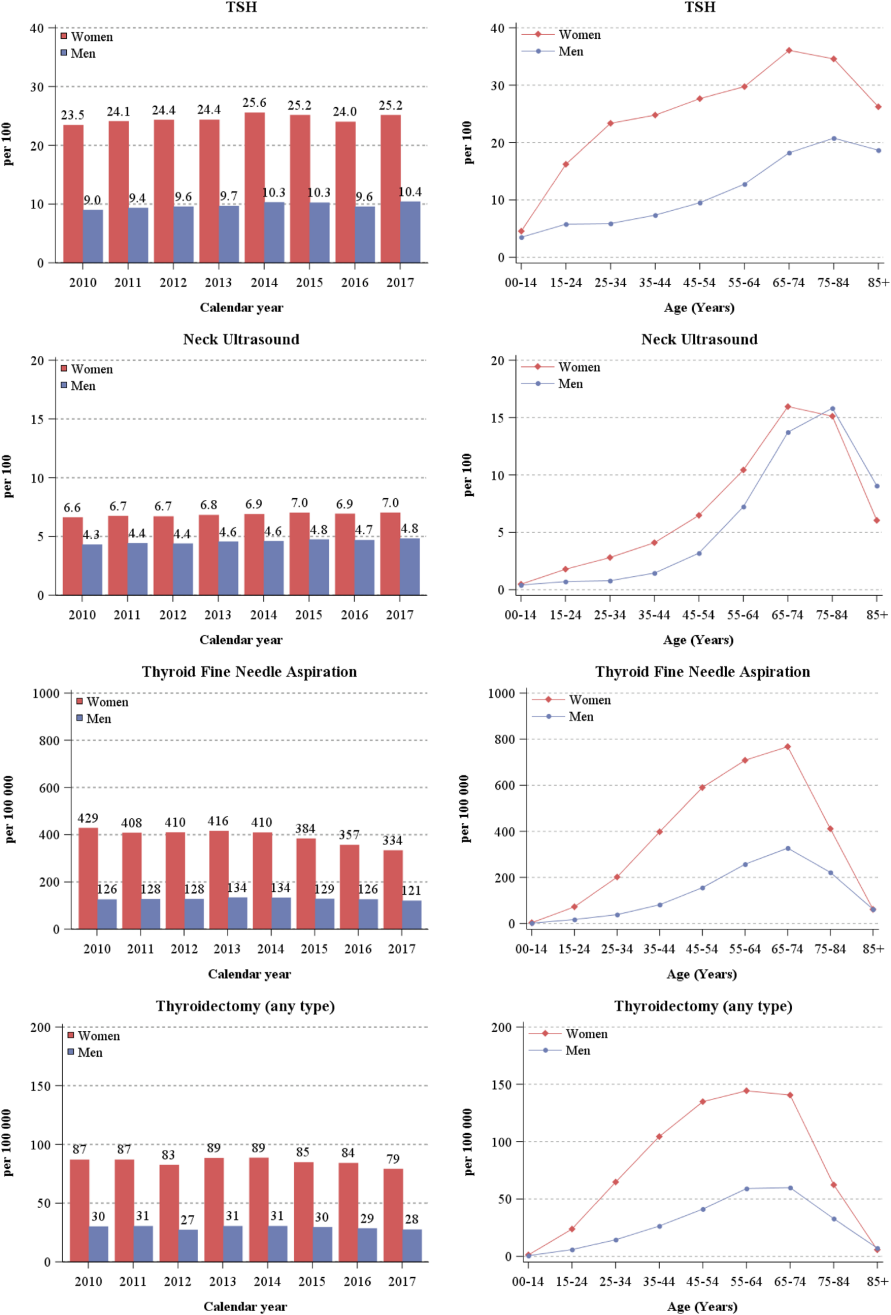
Rates of TSH, neck ultrasound, thyroid fine needle aspiration, and thyroidectomy (any type) by calendar year, sex, and age group. North-eastern Italy, 2010–2017

Between 2010 and 2017, 24.5% of women and 9.8% of men received at least one prescription for TSH testing each year (Table 1). No substantial variations over time were found in either men or women (Fig. 2). The frequency of TSH testing reached > 20% of women aged 25 years or older, with values exceeding 35% at 65–74 years. A more gradual increase with age was found for men, among whom TSH testing was performed > 10% yearly after 65 years and > 20% in those aged 75–84 years. The only remarkable geographical variation was found in Trentino where, among women aged 25–34, nearly 40% were tested for TSH yearly, i.e., approximately two-fold more than in other areas (Online Appendix 2).

Neck ultrasound examinations were performed at least once per year in 6.9% of women and 4.6% of men (Table 1). The peak of neck ultrasounds occurred at ages 65–74 years in women (16%) and 75–84 years in men (15%) (Fig. 2). The proportion of individuals undergoing neck ultrasound slightly increased between 2010 and 2017 (i.e., from 6.6 to 7.0% in women and from 4.3 to 4.8% in men) with a nearly two-fold higher frequency in Emilia-Romagna and Trentino than in Alto Adige (Table 1 and Online Appendix 2).

Thyroid FNA were threefold more frequent in women than men (394 and 128 per 100,000, respectively) with a seven-fold variation between the highest (i.e., Emilia-Romagna) and lowest (Alto Adige) frequency areas in women. Corresponding variation in men was 2.5-fold (Table 1). Women-to-men ratio was consistent in different age groups with a peak at 65–74 years in both sexes (767 in women and 327 per 100,000 in men) (Fig. 2). In women, a consistent decrease of FNA emerged throughout the study period, from 429 in 2010 to 334 per 100,000 in 2017, with a decrease > 6% per year after 2014. In men, 134 per 100,000 FNA were reported yearly in 2013–2014, but they declined to 121 FNA per 100,000 in 2017.

**Fig. 3:**
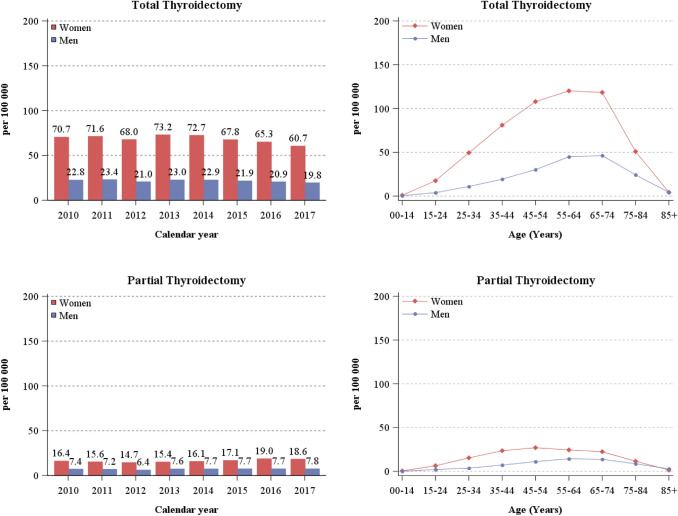
Rates of total and partial thyroidectomies by calendar year, sex, and age group. North-eastern Italy, 2010–2017

The annual number of thyroidectomies was approximately three-fold higher in women (85 per 100,000) than in men (29 per 100,000) (Table 1), with a tendency to decline after 2014. Thyroidectomies increased with age, reaching a plateau at 45–74 years in women and at 55–74 years in men (Fig. 2). Total thyroidectomies were 71 per 100,000 women in 2010, 73 per 100,000 in 2013 and 2014, decreasing to 61 per 100,000 women in 2017 (Fig. 3). Partial thyroidectomies were 16 per 100,000 women in 2010 and 19 per 100,000 women in 2017. Men also showed a decrease in the frequency of total thyroidectomies from 2013 to 2017 (23 and 20 per 100,000 men, respectively) and an increase of partial thyroidectomies from 2012 to 2017 (6.4 and 7.8 per 100,000 men). Notably, the frequency of thyroidectomies was also two-fold higher in Emilia Romagna than in Trentino in both sexes (Online Appendix 2). In women, total thyroidectomies represented more than 75% of all thyroidectomies throughout the examined period, with one partial thyroidectomy every 4.3 total thyroidectomies in 2010, increasing to 4.8 in 2013 (83%), but declining to one partial thyroidectomy every 3.3 total thyroidectomies in 2017 (77%). In men, 3.3 total thyroidectomies were performed for every partial thyroidectomy in 2011 (77%) and 2.5 in 2017 (71%).

**Fig. 4:**
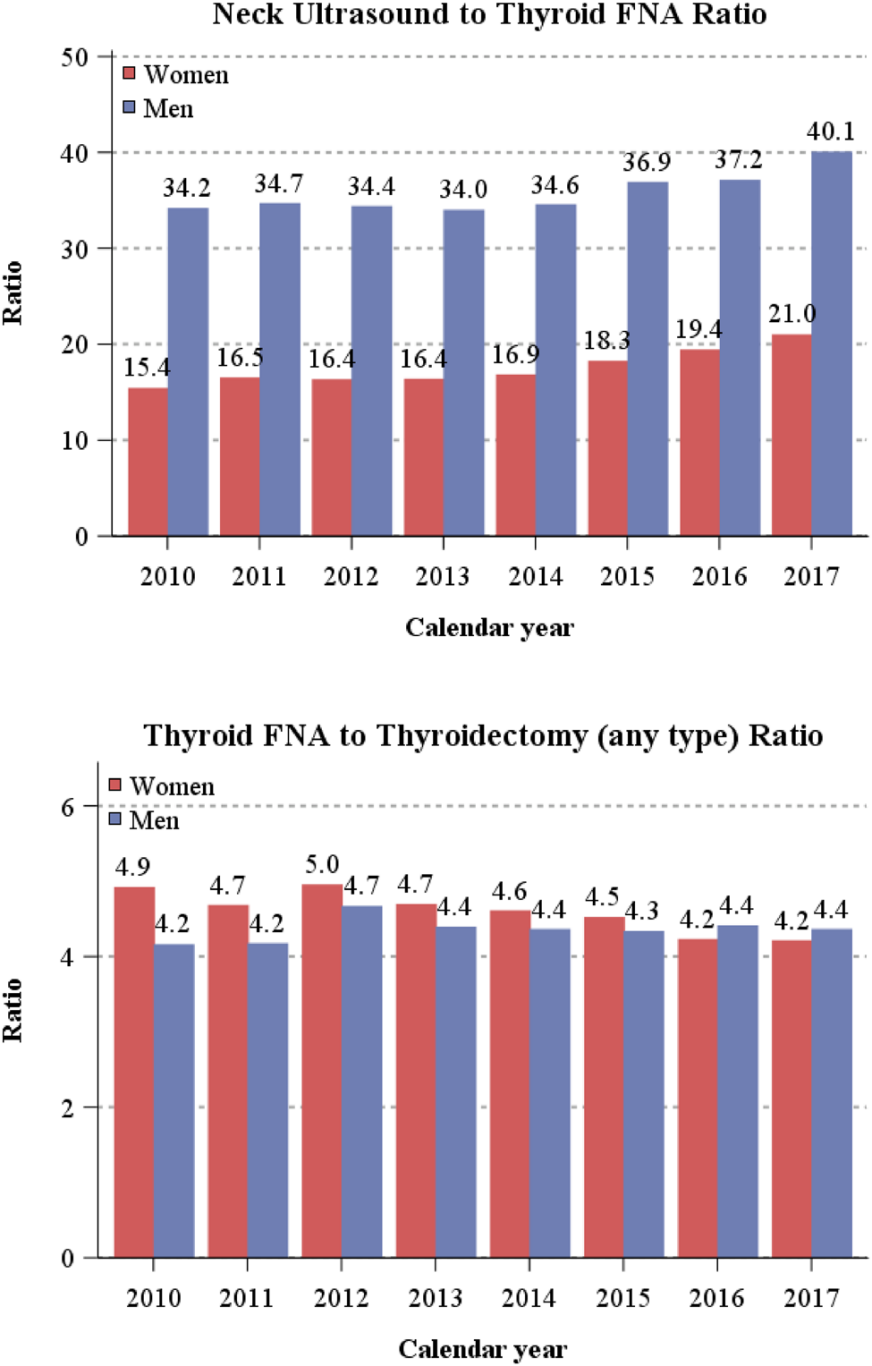
Number of neck ultrasound for every thyroid fine needle aspiration (FNA) and number of thyroid FNA for every thyroidectomy (any type) by calendar year and sex. North-eastern Italy, 2010–2017

The relative frequency of selected procedures was also shown as the ratio of neck ultrasound to FNA and FNA to thyroidectomy (Fig. 4). In women, one FNA was performed every 15.4 neck ultrasound examinations in 2010, increasing to 21.0 in 2017. In men this ratio was two-fold higher, one FNA was performed every 34 neck ultrasound examinations in 2010 and every 40 in 2017. The FNA to thyroidectomy ratios were similar in men and women (i.e., approximately one thyroidectomy every 4.5 FNA), showing negligible variations over time.

## Discussion

This study provides the first population-based evaluation of annual rates of thyroid function testing, neck ultrasound, FNA, and thyroidectomies in 2010–2017 in North-eastern Italy, an area with nearly 12 million inhabitants, 20% of the Italian population.

We found that a large proportion of individuals living in North-eastern Italy underwent thyroid function testing. The percentages of individuals who were tested for TSH at least once per year (25% in women and 10% in men) were consistent with those reported in other countries, such as France [14], Canada [35], UK [36], and Australia [10]. Particularly high frequency of TSH testing was reported in women in Trentino (31% overall and 33% since age 25–34 years, Online Appendix 2), providing confirmation of local variability of thyroid function testing shown elsewhere [36].

Our indirect estimates of the proportion of individuals who received treatments for hypo- (4.1%) or hyperthyroidism (0.23%) in 2017 (4.3% overall) were similar to those obtained elsewhere using a population-based design. In the United Kingdom, the prevalence of treated hypothyroidism increased from 2.3% in 2005 to 3.5% in 2014 [37]. A survey in Spain showed that 4.2% of the population was treated for hypothyroidism and 0.8% for hyperthyroidism [38], while in the USA thyroid hormones (i.e., levotyroxine) were prescribed to 6.4% of adults in 2011–2012 [39]. The association between thyroid dysfunctions and thyroid cancers has received increasing attention [40], but a causal link with thyroid cancer has been firmly established only for nodules [41], while it is still controversial for goiter, hypo- or hyperthyroidism [42].

The frequency of annual neck ultrasound examinations we found (7% in women and 5% in men) was even higher than that reported in other studies, i.e., 2–3% in France [14], 1.5% in USA [13], and 1% in Canada [43]. Of note, thyroid nodules are often incidental findings as neck ultrasound is performed for the examination of carotid arteries (i.e., diseases unrelated to thyroid) and there was a two-fold higher frequency of thyroid FNA per neck ultrasound in women than men.

Our estimates of annual FNA (128 per 100,000 men and 394 per 100,000 women) are also higher than those reported in the only comparable population-based study in France (i.e., approximately 200 FNA per 100,000 population, men and women combined) [14]. Interestingly, FNA is the examination that shows the largest variation (i.e., > two-fold) across study areas. The decrease of FNA in the study period was more marked in the study area with the highest yearly rates (i.e., Emilia Romagna, Online Appendix 2). Information on overall reduction of FNA with substantially unchanged rates of neck ultrasound may suggest of a more stringent selection of nodules that needed further examination during the last study years.

The number of thyroidectomies in North-eastern Italy for any indication between 2010 and 2017 (29 per 100,000 men and 85 per 100,000 women per year, 58 per 100,000 overall) was similar to the results reported in France [14] and the United States [25], until 2012 and 2014. Notably, in our study the frequency of total thyroidectomies has shown a downward trend since 2015, probably as a consequence of the reduction of FNA since 2015 and according to the most recent guidelines recommending that only > 1 cm nodules should be evaluated, since they have a greater potential to be clinically significant cancers [5]. In addition, after 2015 we observed a more conservative surgery approach with low but increasing rates of partial thyroidectomies. These findings could mirror, to some extent, the cultural trend induced by the worldwide steep increase of papillary microcarcinoma, in favor of a less aggressive diagnostic attitude towards small thyroid tumors. This trend culminated in the contents of 2015 ATA guidelines [5], which include a recommendation against FNA for < 10 mm diameter thyroid nodules, even when suspicious on ultrasound basis. The ATA guidelines were preceded by meeting sessions, editorials as well as review articles which anticipated this less aggressive vision [5].

Italy is one of the countries where the burden of thyroid cancer incidence and overdiagnosis is among the largest worldwide [19, 26] and the areas in the present study include the highest and lowest thyroid cancer incidence rates in Italy [26]. In North-eastern Italy, persons living after a thyroid cancer diagnosis were 0.2% of the overall population in 2010 [44], most of them cured [45] but still needing lifelong treatments. Age-adjusted incidence rates in 2010–2012 in South Tyrol were 11 per 100,000 women and 5 per 100,000 men, while nearly threefold higher rates were found in Emilia Romagna (i.e., 24 per 100,000 women and 15 per 100,000 men) [26]. Among the indicators of diagnostic pressure evaluated in the study, the strongest correlation with thyroid cancer incidence was found for FNA rates (Spearman correlation > 0.7 in men and women). In women, a strong correlation also emerged for neck ultrasound (Spearman 0.76), consistently with results in South Korea, where screening is the most important driver of the epidemic of thyroid cancer, particularly among females [16]. The frequency of ultrasonography for thyroid screening reached coverage > 15% in some South Korean regions and is 3- to 4-fold higher than the frequency of neck ultrasound recorded in our study. No favourable effect of screening on thyroid cancer mortality emerged [16, 20].

Strengths of the presents study include the population-based design, the inclusion of a substantial part of Italian population, and the availability of data until 2017. Study limitations are the lack of linkage individuals for different tests performed, and of information on the determinants of examinations and their appropriateness according to current guidelines. In addition, we could not assess the outcomes of examinations performed.

Our results are not meant to recommend ideal testing frequency for TSH, neck ultrasound, or thyroid FNA in our, as well as in other populations [8]. Several comorbidities other than known or suspected thyroid diseases may have prompted TSH testing (e.g., type 2 diabetes or hypertension) [11] and neck ultrasound examination (e.g., carotid stenosis). Recently, patterns of TSH testing, the most commonly prescribed thyroid examination by clinicians, have been explored in Canada [46], showing a relatively high proportion (22%) of TSH testing not conforming to current test-ordering guidelines. Moreover, benign thyroid nodules can be safely followed with less intense protocols than those proposed in the past years [47].

In the paucity of population-based data on the appropriateness of current practice of thyroid clinical impact of test overuse or underuse [11, 48], future studies linking individual number of different thyroid examinations would be of great interest, particularly in specific population subgroups (women < 25 years, the elderly population). These studies would further increase the knowledge on the pathways to the diagnosis of thyroid cancer and other thyroid disorders, to differentiate clinically relevant diseases from those with no impact on mortality [19, 49]. In addition, they would contribute to evaluate adherence to evolving guidelines [5, 7, 50] and they should be used to reduce unnecessary medicalization of patients [51] and unnecessary costs for the healthcare system [23, 52, 53].

## Conclusion

Variations in the frequency of thyroid examination across otherwise rather similar Italian regions suggest the need for a reappraisal of the indications for the detection and management of thyroid disorders. There is certainly room for a reduction of FNA examinations in some areas. According to growing evidence suggesting the feasibility of more conservative surgical approaches [54], indications to total thyroidectomy may be reconsidered. Most of all, the evidence of a first impact of recent recommendations [7, 55, 56] on thyroid diagnosis and treatments in real-life clinical practice stresses the need of further dissemination efforts, sharing, and implementation of emerging evidence-based guidelines.

## Supplementary Information

Below is the link to the electronic supplementary material.

Supplementary file1 (DOCX 473 KB)

## Data Availability

Dataset supporting our findings is available, upon reasonable request, by the corresponding author.

## References

[1] Golden SH, Robinson KA, Saldanha I, Anton B, Ladenson PW (2009). Clinical review: prevalence and incidence of endocrine and metabolic disorders in the United States: a comprehensive review. J Clin Endocrinol Metab.

[2] Garmendia Madariaga AG, Santos Palacios S, Guillén-Grima F, Galofré JC (2014). The incidence and prevalence of thyroid dysfunction in Europe: a meta-analysis. J Clin Endocrinol Metab.

[3] Walsh JP (2016). Managing thyroid disease in general practice. Med J Aust.

[4] Taylor PN, Albrecht D, Scholz A, Gutierrez-Buey G, Lazarus JH, Dayan CM (2018). Global epidemiology of hyperthyroidism and hypothyroidism. Nat Rev Endocrinol.

[5] Haugen BR, Alexander EK, Bible KC, Doherty GM, Mandel SJ, Nikiforov YE (2016). 2015 American Thyroid Association management guidelines for adult patients with thyroid nodules and differentiated thyroid cancer: The American Thyroid Association Guidelines Task Force on thyroid nodules and differentiated thyroid cancer. Thyroid.

[6] Gharib H, Papini E, Garber JR, Duick DS, Harrell RM, Hegedüs L (2016). Medical guidelines for clinical practice for the diagnosis and management of thyroid nodules-2016 update. Endocr Pract.

[7] Pacini F, Basolo F, Bellantone R, Boni G, Cannizzaro MA, De Palma M (2018). Italian consensus on diagnosis and treatment of differentiated thyroid cancer: joint statements of six Italian societies. J Endocrinol Investig.

[8] Hall SF, Webber C, Groome PA, Booth CM, Nguyen P, DeWit Y (2019). Do doctors who order more routine medical tests diagnose more cancers? A population-based study from Ontario Canada. Cancer Med.

[9] IARC Expert Group on Thyroid Health Monitoring after Nuclear Accidents (2018) Thyroid health monitoring after nuclear accidents, vol 46. IARC Technical Publications, Lyon

[10] Hong A, Stokes B, Otahal P, Owens D, Burgess JR (2017). Temporal trends in thyroid-stimulating hormone (TSH) and thyroid peroxidase antibody (ATPO) testing across two phases of iodine fortification in Tasmania (1995–2013). Clin Endocrinol (Oxf).

[11] Werhun A, Hamilton W (2015). Thyroid function testing in primary care: overused and under-evidenced? A study examining which clinical features correspond to an abnormal thyroid function result. Fam Pract.

[12] Brito JP, Al Nofal A, Montori VM, Hay ID, Morris JC (2015). The impact of subclinical disease and mechanism of detection on the rise in thyroid cancer incidence: a population-based study in olmsted county, minnesota during 1935 through 2012. Thyroid.

[13] Haymart MR, Banerjee M, Reyes-Gastelum D, Caoili E, Norton EC (2019). Thyroid ultrasound and the increase in diagnosis of low-risk thyroid cancer. J Clin Endocrinol Metab.

[14] Mathonnet M, Cuerq A, Tresallet C, Thalabard JC, Fery-Lemonnier E, Russ G (2017). What is the care pathway of patients who undergo thyroid surgery in France and its potential pitfalls? A national cohort. BMJ Open.

[15] Vaccarella S, Dal Maso L, Laversanne M, Bray F, Plummer M, Franceschi S (2015). The impact of diagnostic changes on the rise in thyroid cancer incidence: a population-based study in selected high-resource countries. Thyroid.

[16] Ahn HS, Kim HJ, Kim KH, Lee YS, Han SJ, Kim Y (2016). Thyroid cancer screening in South Korea increases detection of papillary cancers with no impact on other subtypes or thyroid cancer mortality. Thyroid.

[17] Lin JS, Bowles EJA, Williams SB, Morrison CC (2017). Screening for thyroid cancer: updated evidence report and systematic review for the US preventive services task force. JAMA.

[18] Lortet-Tieulent J, Franceschi S, Dal Maso L, Vaccarella S (2019). Thyroid cancer "epidemic" also occurs in low- and middle-income countries. Int J Cancer.

[19] Li M, Dal Maso L, Vaccarella S (2020). Global trends in thyroid cancer incidence and the impact of overdiagnosis. Lancet Diabetes Endocrinol.

[20] Jun JK, Hwang SY, Hong S, Suh M, Choi KS, Jung KW (2020). Association of screening by thyroid ultrasonography with mortality in thyroid cancer: a case-control study using data from two national surveys. Thyroid.

[21] Colbeth HL, Genere N, Hall CB, Jaber N, Brito JP, El Kawkgi OM (2020). Evaluation of medical surveillance and incidence of post-September 11, 2001, thyroid cancer in world trade center-exposed firefighters and emergency medical service workers. JAMA Intern Med.

[22] Sosa JA, Hanna JW, Robinson KA, Lanman RB (2013). Increases in thyroid nodule fine-needle aspirations, operations, and diagnoses of thyroid cancer in the United States. Surgery.

[23] Ho TW, Shaheen AA, Dixon E, Harvey A (2011). Utilization of thyroidectomy for benign disease in the United States: a 15-year population-based study. Am J Surg.

[24] Pelizzo MR, Rubello D, Bernardi C, Gemo G, Bertazza L, Schievano E (2014). Thyroid surgical practices shaping thyroid cancer incidence in North-Eastern Italy. Biomed Pharmacother.

[25] Francis DO, Randolph G, Davies L (2017). Nationwide variation in rates of thyroidectomy among US medicare beneficiaries. JAMA Otolaryngol Head Neck Surg.

[26] Dal Maso L, Panato C, Franceschi S, Serraino D, Buzzoni C, Busco S (2018). The impact of overdiagnosis on thyroid cancer epidemic in Italy, 1998–2012. Eur J Cancer.

[27] Lise M, Franceschi S, Buzzoni C, Zambon P, Falcini F, Crocetti E (2012). Changes in the incidence of thyroid cancer between 1991 and 2005 in Italy: a geographical analysis. Thyroid.

[28] Busco S, Giorgi Rossi P, Sperduti I, Pezzotti P, Buzzoni C, Pannozzo F (2013). Increased incidence of thyroid cancer in Latina, Italy: a possible role of detection of subclinical disease. Cancer Epidemiol.

[29] Van den Bruel A, Francart J, Dubois C, Adam M, Vlayen J, De Schutter H (2013). Regional variation in thyroid cancer incidence in Belgium is associated with variation in thyroid imaging and thyroid disease management. J Clin Endocrinol Metab.

[30] Panato C, Serraino D, De Santis E, Forgiarini O, Angelin T, Bidoli E (2019). Thyroid cancer in Friuli Venezia Giulia, northeastern Italy: incidence, overdiagnosis, and impact of type of surgery on survival. Tumori J.

[31] Rahman ST, McLeod DSA, Pandeya N, Neale RE, Bain CJ, Baade P (2019). Understanding pathways to the diagnosis of thyroid cancer: are there ways we can reduce over-diagnosis?. Thyroid.

[32] Ministero della Salute. Il nuovo nomenclatore: DPCM 12 gennaio 2017. http://www.salute.gov.it/portale/temi/p2_6.jsp?lingua=italiano&id=1767&area=programmazioneSanitariaLea&menu=vuoto. Accessed 4 Nov 2020

[33] Centers for Disease Control and Prevention. International Classification of Diseases, 9th Revision, Clinical Modification (ICD-9-CM). https://www.cdc.gov/nchs/icd/icd9cm.htm. Published 2016. Accessed 4 Nov 2020

[34] WHO Collaborating Centre for Drug Statistics Methodology (2018) ATC classification index with DDDs, 2019. Oslo, Norway

[35] Wintemute K, Greiver M, McIsaac W, Del Giudice ME, Sullivan F, Aliarzadeh B (2019). Choosing Wisely Canada campaign associated with less overuse of thyroid testing: retrospective parallel cohort study. Can Fam Phys.

[36] Vaidya B, Ukoumunne OC, Shuttleworth J, Bromley A, Lewis A, Hyde C (2013). Variability in thyroid function test requests across general practices in south-west England. Qual Prim Care.

[37] Razvi S, Korevaar TIM, Taylor P (2019). Trends, determinants, and associations of treated hypothyroidism in the United Kingdom, 2005–2014. Thyroid.

[38] Valdés S, Maldonado-Araque C, Lago-Sampedro A, Lillo JA, Garcia-Fuentes E, Perez-Valero V (2017). Population-based national prevalence of thyroid dysfunction in spain and associated factors: diabetes study. Thyroid.

[39] Kantor ED, Rehm CD, Haas JS, Chan AT, Giovannucci EL (2015). Trends in prescription drug use among adults in the United States from 1999–2012. JAMA.

[40] Tran TV, Kitahara CM, de Vathaire F, Boutron-Ruault MC, Journy N (2020). Thyroid dysfunction and cancer incidence: a systematic review and meta-analysis. Endocr Relat Cancer.

[41] Franceschi S, Preston-Martin S, Dal Maso L, Negri E, La Vecchia C, Mack WJ (1999). A pooled analysis of case-control studies of thyroid cancer. IV. Benign thyroid diseases. Cancer Causes Control.

[42] Rinaldi S, Plummer M, Biessy C, Tsilidis KK, Østergaard JN, Overvad K (2014). Thyroid-stimulating hormone, thyroglobulin, and thyroid hormones and risk of differentiated thyroid carcinoma: the EPIC study. J Natl Cancer Inst.

[43] Hall SF, Irish J, Groome P, Griffiths R (2014). Access, excess, and overdiagnosis: the case for thyroid cancer. Cancer Med.

[44] AIRTUM Working Group (2014). Italian cancer figures, report 2014: prevalence and cure of cancer in Italy. Epidemiol Prev.

[45] Dal Maso L, Panato C, Guzzinati S, Serraino D, Francisci S, Botta L (2019). Prognosis of long-term cancer survivors: a population-based estimation. Cancer Med.

[46] Birk-Urovitz E, Del Giudice M, Meaney C, Grewal K (2017). Use of thyroid-stimulating hormone tests for identifying primary hypothyroidism in family medicine patients. Can Fam Phys..

[47] Grani G, Lamartina L, Biffoni M, Giacomelli L, Maranghi M, Falcone R (2018). Sonographically estimated risks of malignancy for thyroid nodules computed with five standard classification systems: changes over time and their relation to malignancy. Thyroid.

[48] O'Sullivan JW, Albasri A, Nicholson BD, Perera R, Aronson JK, Roberts N (2018). Overtesting and undertesting in primary care: a systematic review and meta-analysis. BMJ Open.

[49] Journy NMY, Bernier MO, Doody MM, Alexander BH, Linet MS, Kitahara CM (2017). Hyperthyroidism, hypothyroidism, and cause-specific mortality in a large cohort of women. Thyroid.

[50] Russ G, Bonnema SJ, Erdogan MF, Durante C, Ngu R, Leenhardt L (2017). European Thyroid Association guidelines for ultrasound malignancy risk stratification of thyroid nodules in adults: the EUTIRADS. Eur Thyroid J.

[51] Jensen CB, Saucke MC, Francis DO, Voils CI, Pitt SC (2020). From overdiagnosis to overtreatment of low-risk thyroid cancer: a thematic analysis of attitudes and beliefs of endocrinologists, surgeons, and patients. Thyroid.

[52] Lubitz CC, Kong CY, McMahon PM, Daniels GH, Chen Y, Economopoulos KP (2014). Annual financial impact of well differentiated thyroid cancer care in the United States. Cancer.

[53] Kiel S, Ittermann T, Völzke H, Chenot JF, Angelow A (2020). Frequency of thyroid function tests and examinations in participants of a population-based study. BMC Health Serv Res.

[54] Molinaro E, Campopiano MC, Pieruzzi L, Matrone A, Agate L, Bottici V (2020). Active surveillance in papillary thyroid microcarcinomas is feasible and safe: experience at a single Italian center. J Clin Endocrinol Metab.

[55] Tessler FN, Middleton WD, Grant EG, Hoang JK, Berland LL, Teefey SA (2017). ACR thyroid imaging, reporting and data system (TI-RADS): white paper of the ACR TI-RADS Committee. J Am Coll Radiol.

[56] Lamartina L, Durante C, Lucisano G, Grani G, Bellantone R, Lombardi CP (2017). Are evidence-based guidelines reflected in clinical practice? An analysis of prospectively collected data of the Italian Thyroid Cancer Observatory. Thyroid.

