# Supplementary material for: Trends in thyroid function testing, neck ultrasound, thyroid fine needle aspiration, and thyroidectomies in North-eastern Italy
Source: J Endocrinol Invest. 2021 Jan 18;44(8):1679–88. doi: 10.1007/s40618-020-01475-3 (PMC8285305; doi:10.1007/s40618-020-01475-3)
Supplement: Supplementary file 1 — Supplementary file1 (DOCX 473 KB) [file 40618_2020_1475_MOESM1_ESM.docx]

**Appendix 1. Administrative codes to derive procedures. North-eastern Italy, 2010-2017**

| **Procedure** | **Code** | **Description** | **Database** |
| --- | --- | --- | --- |
| TSH | 90.42.1 | Thyroid-stimulating hormone (thyrotropin) (TSH) | Outpatient Services  (Prestazioni ambulatoriali) |
| NECK ULTRASOUND | 88.71.4 | Diagnostic Ultrasound Procedures of the Head and Neck |  |
|  | 88.71.5 | ECO(COLOR)DOPPLER: Transcranial |  |
|  | 88.73.5 | ECO(COLOR)DOPPLER: VASCULAR, NECK |  |
| FINE NEEDLE ASPIRATION***** | 06.01 | Ultrasound-Guided Fine Needle Aspiration of Thyroid |  |
|  | 06.01.1 | Fine Needle Aspiration of Thyroid |  |
|  | 06.11.1 | Biopsy of Thyroid |  |
|  | 06.11.2 | Ultrasound-Guided Biopsy of Thyroid |  |
| SURGERY |  |  | Hospital Discharge  (Schede di dimissione ospedaliera, SDO) |
|  | 06.4, 06.50, 06.52 | Total Thyroidectomy |  |
|  | 06.2, 06.3x, 06.51 | Partial Thyroidectomy |  |
| DRUGS | H03 | H03AA (Levotiroxine) H03BB (Thiamazole) | Drug Prescriptions |

*Derived also from Hospital Discharge database

**Appendix 2. People to whom drugs for hypothyroidism or hyperthyroidism^1^ were prescribed, rates of TSH, neck ultrasound, thyroid fine needle aspiration, and thyroidectomy (any type) by sex, region, and age group. North-eastern Italy, 2010-2017**


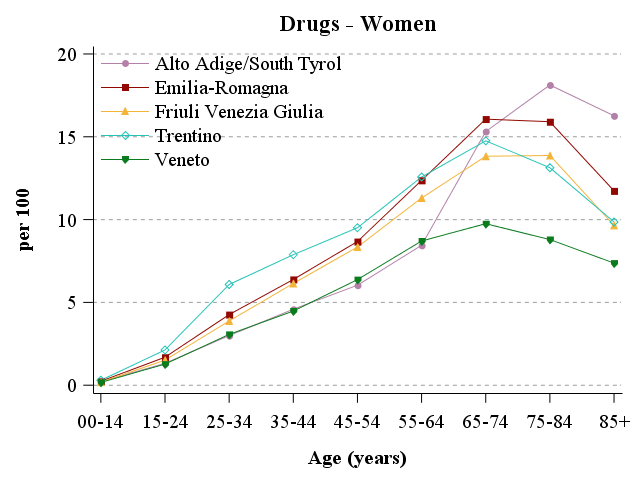

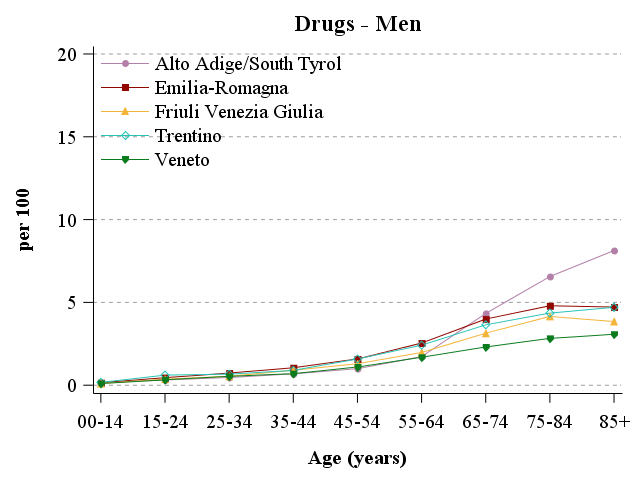


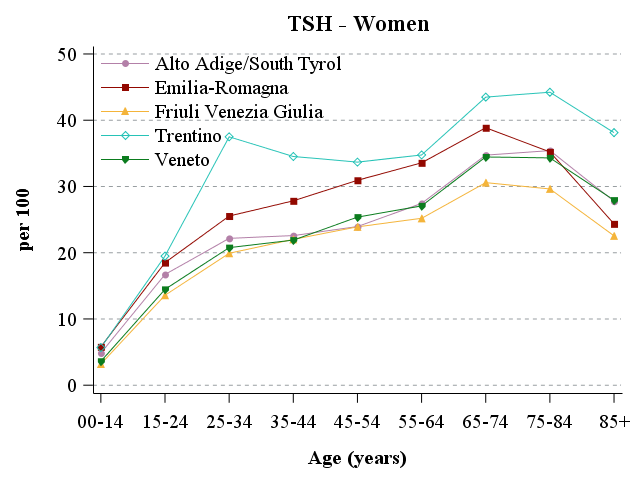

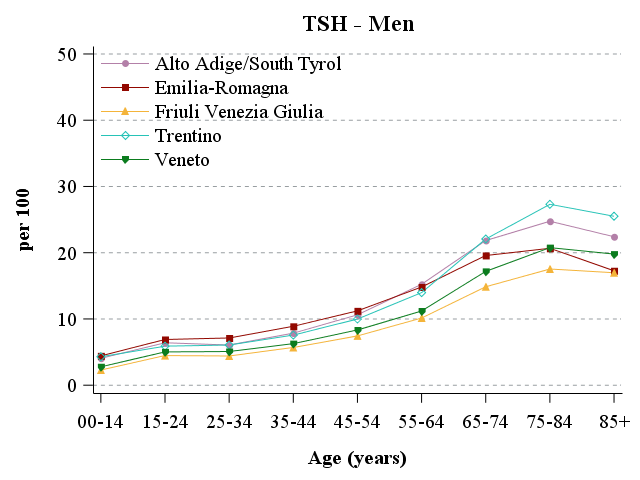


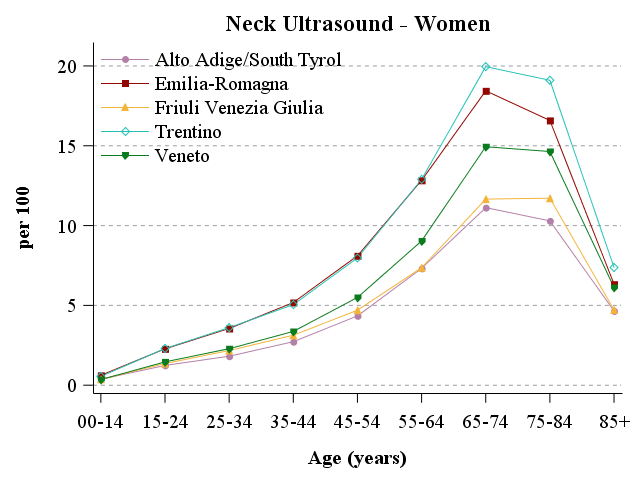

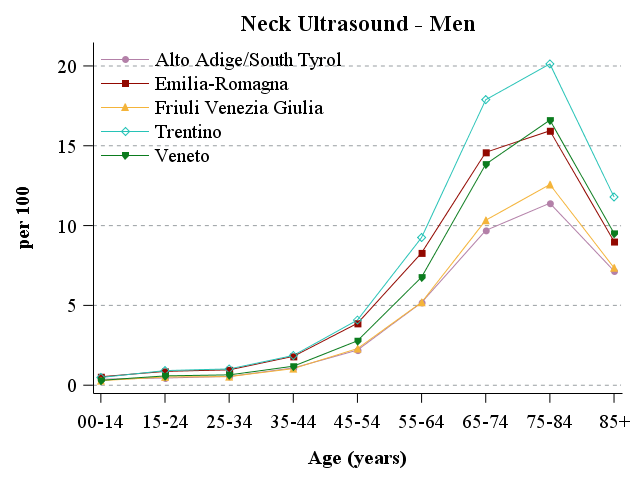


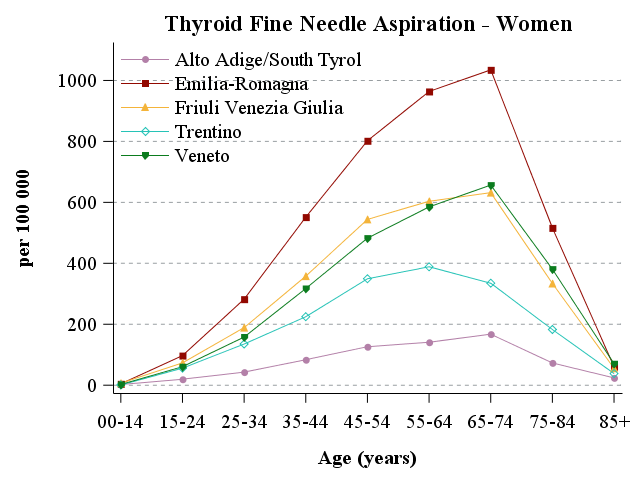

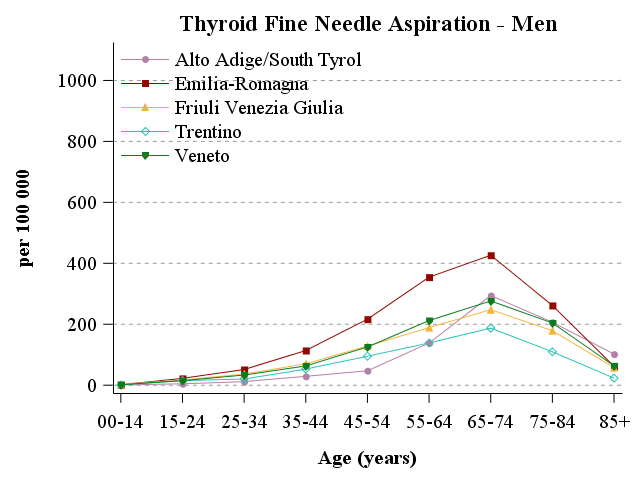


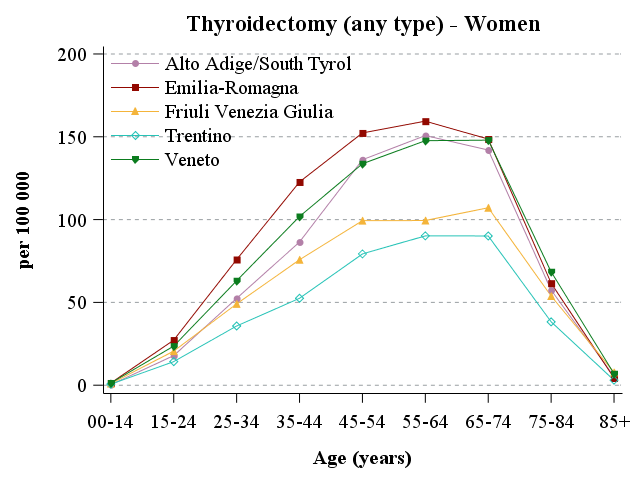

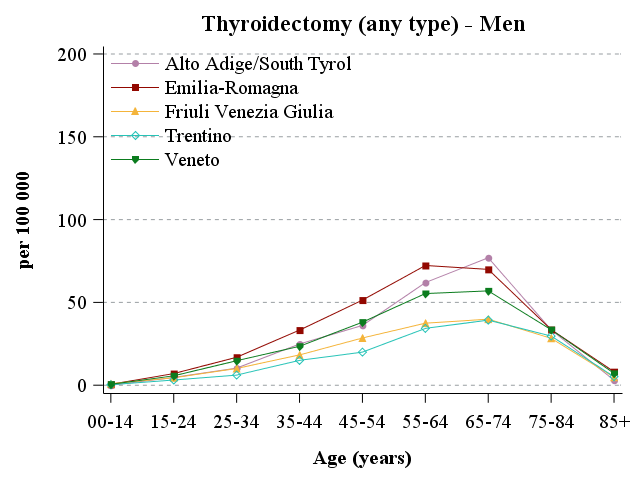


**^1^** At least two drug prescriptions in 2017
